# Carbon Micro-Alloying Promotes Creep Flow via Enhanced Structural Heterogeneity in Fe-Based Amorphous Alloys

**DOI:** 10.3390/ma18194637

**Published:** 2025-10-09

**Authors:** Deyu Cao, Sishi Teng, Jiajie Lv, Xin Su, Yu Tong, Mingliang Xiang, Lijian Song, Meng Gao, Yan Zhang, Juntao Huo, Junqiang Wang

**Affiliations:** 1 School of Materials Science and Engineering, Zhejiang University of Technology, Hangzhou 310014, China; caodeyu@nimte.ac.cn; 2 Zhejiang Key Laboratory of Magnetic Materials and Applications, Ningbo Institute of Materials Technology and Engineering, Chinese Academy of Sciences, Ningbo 315201, China; tengsishi@nimte.ac.cn (S.T.); lvjiajie@nimte.ac.cn (J.L.); suxin@nimte.ac.cn (X.S.); songlj@nimte.ac.cn (L.S.); gaomeng@nimte.ac.cn (M.G.); yzhang@nimte.ac.cn (Y.Z.); huojuntao@nimte.ac.cn (J.H.)

**Keywords:** metallic glasses, amorphous alloys, nanoindentation creep, structural heterogeneity

## Abstract

Tuning structural heterogeneity in metallic glasses is key to improving their mechanical performance. Here we examine how carbon micro-alloying modulates the relaxation dynamics and creep of Fe-based amorphous ribbons. Increasing carbon content lowers the crystallization temperature, amplifies *β*-relaxation, and reduces hardness, consistent with enhanced atomic mobility. Nanoindentation creep, fitted with a stretched-exponential model, shows a decreasing exponent with carbon addition, indicating broader relaxation–time distributions and stronger dynamic heterogeneity. Nanoscale force-mapping further reveals a larger fraction of liquid-like regions and pronounced viscoelastic heterogeneity in carbon-rich samples. These changes facilitate the activation of shear-transformation zones and promote room-temperature creep flow. Together, the results establish a direct link between structural heterogeneity, relaxation processes, and mechanical response, providing guidance for the design of ductile metallic glasses.

## 1. Introduction

Metallic glasses (MGs), also known as amorphous alloys, exhibit exceptional combinations of high strength, large elastic limit, and excellent soft magnetic properties due to their disordered atomic structures and absence of long-range periodicity [1,2,3]. However, their practical applications are often hindered by limited plasticity at room temperature, primarily resulting from highly localized deformation and the lack of dislocation-mediated mechanisms [3]. Recent advances have demonstrated that atomic-scale structural tuning through micro-alloying offers an effective approach to modulate the plastic flow behavior of MGs, providing a promising pathway to mitigate their intrinsic brittleness [4]. Notably, element-specific softening has been reported in Fe-based glasses under certain chemistries—for example, W additions that lower hardness in an Fe-based structural amorphous metal [5], and P-rich FeP(C) systems that exhibit lower shear modulus and higher Poisson’s ratio (a softer matrix) [6]; these cases illustrate that composition can decrease strength/hardness depending on solute type and concentration [7,8]. Among candidate solutes, carbon has shown particular promise in Fe-based MGs, where it not only enhances soft magnetic performance [9,10,11] but also improves compressive plasticity in bulk forms [9]. Nevertheless, the mechanisms by which carbon modifies room-temperature deformation behavior remain poorly understood. This challenge arises from the lack of periodicity and the absence of conventional structural defects, which limits the applicability of traditional crystalline models and hinders the establishment of structure–mechanical property relationships.

To address this challenge, it is crucial to understand how atomic-scale structural heterogeneity influences the initiation and evolution of plastic deformation in MGs. Plastic flow in MGs is primarily accommodated through the formation and propagation of shear bands, which originate from localized atomic rearrangements within shear transformation zones (STZs) [1,2,3,12,13,14,15]. These STZs are rooted in the intrinsic nanoscale heterogeneity of MGs [16,17,18,19,20], where densely packed solid-like regions coexist with loosely packed, liquid-like regions. The liquid-like regions exhibit higher atomic mobility and serve as the structural origin of STZs, acting as fundamental carriers of plastic deformation. Upon mechanical loading, these regions are preferentially activated, initiating localized shear events that accommodate plastic flow. The degree of structural heterogeneity plays a critical role in determining mechanical behavior, particularly room-temperature ductility, as a higher fraction of liquid-like regions enhances the activation of STZs, promotes more uniform plastic deformation, and delays the onset of catastrophic failure [1,2,21,22]. However, a systematic understanding of how carbon content regulates nanoscale heterogeneity and its role in governing plastic flow is still lacking.

Bridging this structural complexity with macroscopic behavior, relaxation dynamics provide a powerful framework for understanding the evolution of structural heterogeneity and its role in plastic flow. The metastable nature of amorphous alloys gives rise to a complex energy landscape, where thermally or mechanically driven atomic motions manifest as coupled relaxation modes. Typically, three distinct relaxation processes are observed: fast relaxation, *β*-relaxation, and *α*-relaxation [1,2,23]. At the microscopic level, fast relaxation is attributed to the chain-like motion of liquid-like atoms [23,24], *β*-relaxation arises from the activation of atoms in localized liquid-like regions [25,26,27], and *α*-relaxation corresponds to irreversible structural rearrangements associated with large-scale atomic mobility [2,28]. Numerous studies have demonstrated that these relaxation dynamics are closely linked to macroscopic properties. For instance, stress-induced *α*-relaxation is strongly related to yield behavior [12,28,29], while *β*-relaxation influences various material properties, including plasticity, magnetoelastic coupling, glass transition, crystallization, and thermal stability [1,2,3,26]. Thus, investigating how micro-alloying influences relaxation dynamics offers key insights into the interplay between structural heterogeneity and mechanical properties in amorphous alloys.

To address how carbon micro-alloying regulates nanoscale heterogeneity and its impact on room-temperature creep, this study pursues two objectives: (i) quantify how carbon content modifies the *β*-relaxations and viscoelastic heterogeneity; (ii) establish the composition-dependent trend between dynamic heterogeneity and nanoindentation creep. Accordingly, we systematically investigate the structural, thermal, and mechanical responses of Fe_82_Si_3.6_B_12.4−x_C_x_Cu_1_Ni_1_ (x = 3, 4 and 5 at. %) amorphous ribbons produced by melt spinning. By precisely varying carbon content, we link compositional tuning to nanoscale heterogeneity, relaxation dynamics, and deformation behavior. Nanoindentation creep tests assess time-dependent plastic flow, while structural heterogeneity is examined alongside β-relaxation and the distribution of nanoscale liquid-like regions that mediate deformation. By establishing clear correlations among mechanical response, relaxation dynamics, and structural inhomogeneity, this work clarifies the origins of carbon-associated softening in Fe-based metallic glasses and provides guidance for designing tougher, more ductile compositions.

## 2. Materials and Experiments

### 2.1. Sample Preparation, Structural and Dynamic Characterization

Alloy ingots with nominal compositions Fe_82_Si_3.6_B_12.4−x_C_x_Cu_1_Ni_1_ (x = 3, 4, and 5 at. %) were prepared by copper-mold casting. For clarity, these compositions are hereafter denoted as C3, C4, and C5, respectively. The selected carbon content range was determined based on its favorable glass-forming ability. In contrast, alloys with lower carbon content (x < 3 at. %) failed to achieve a fully amorphous structure when processed using a laboratory-scale melt-spinning technique. The elemental mixtures were re-melted at a set point of *T*_melt_ ≈ 1200 °C and cycled five times to ensure chemical homogeneity. Alloying and re-melting were performed under an argon atmosphere to suppress oxidation. Thin-ribbon samples, approximately 30 μm in thickness, were subsequently fabricated by single-roller melt spinning onto a copper wheel rotating at a tangential speed of 55 m/s. A consolidated workflow is provided in Figure S1 (Supplementary Materials) for clarity.

Room-temperature X-ray diffraction (XRD) was performed on a Bruker D8 DISCOVER diffractometer (Bruker Scientific LLC, Billerica, MA, USA) using Cu Kα radiation (λ = 1.5406 Å). Patterns were collected over the 2θ range with a step size of 0.02° at a scan rate of 5°·min^−1^, with the tube operated at 40 kV, confirming the amorphous structure of the metallic glasses. Differential scanning calorimetry (DSC) was carried out on a NETZSCH 404C calorimeter (NETZSCH-Gerätebau GmbH, Selb, Germany) at 20 K·min^−1^, and the onset crystallization temperature (*T*_x_) was determined from the heat-flow curves. Dynamic mechanical analysis (DMA) was performed on a TA Instruments Q800 dynamic mechanical analyzer (TA Instruments–Waters LLC, New Castle, DE, USA) to probe atomic mobility in Fe_82_Si_3.6_B_12.4−x_C_x_Cu_1_Ni_1_ (x = 3, 4 and 5) amorphous ribbons. The measurements were carried out at a constant frequency of 1 Hz with a heating rate of 5 K/min, covering a temperature range from room temperature up to 700 K. Data were acquired/processed using DIFFRAC.SUITE (EVA v7.3, Bruker, Billerica, MA, USA), Proteus v9 (NETZSCH), and TRIOS v5.8 (TA Instruments).

### 2.2. Nanoindentation Tests

Nanoindentation experiments were conducted using a Hysitron TI950 nanoindenter (Bruker, Billerica, MA, USA) equipped with a Berkovich diamond tip with an effective radius *R*_tip_ of approximately 100 nm. Microhardness was evaluated using a loading protocol consisting of a ramp to a peak load of 3000 μN at a constant rate of 600 μN·s^−1^, followed by a 2 s hold at maximum load and unloading to zero over 5 s. To assess nanoindentation creep behavior, the maximum load of 3000 μN was maintained for a dwell time of 30 s. To ensure data reliability and reproducibility, each measurement was repeated at eight distinct locations. Prior to tests, the nano-indenter was calibrated using standard fused silica, ensuring reliable force and displacement measurements, particularly critical for accurately capturing time-dependent creep responses. The specimens are free-standing, melt-spun ribbons rather than ribbons on a rigid substrate. During nanoindentation creep holds, the maximum depth was kept well below 10% of the ribbon thickness. Within this regime, substrate effects are negligible for the present geometry.

### 2.3. Atomic Force Microscopy Measurements

Nanoscale heterogeneity of as-cast metallic-glass (MG) ribbons was mapped by AFM (Bruker Dimension Icon) in Peak Force-QNM mode using silicon probes (tip radius ≈3 nm; spring constant ≈ 40 N m^−1^). Deflection sensitivity and cantilever spring constant were calibrated by thermal tuning and verified on a sapphire standard. Freshly exposed surfaces were scanned over 200 × 200 nm^2^ areas at 512 × 512 pixels to obtain simultaneous topography and adhesion maps. At each pixel, a full force–distance curve was acquired; adhesion was taken from the retract branch, and local compliance/reduced modulus was extracted by standard contact-mechanics fits (DMT/Sneddon) [27,30,31,32]. Measurements were performed under ultra-clean conditions at 23 ± 1 °C and 20% relative humidity.

## 3. Results and Discussion

### 3.1. Typical Physical Parameters

**Figure 1:**
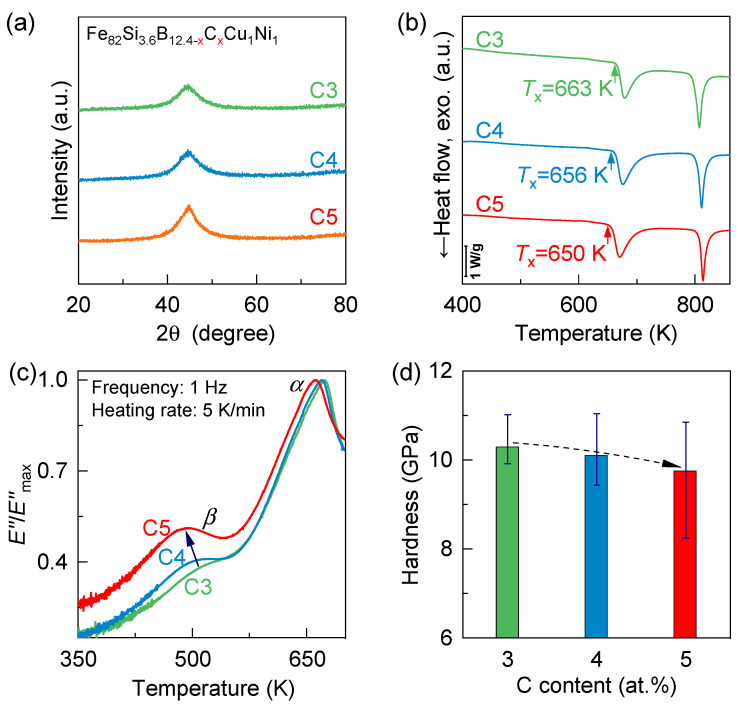
Effects of micro-alloying with carbon (C) on typical physical parameters of Fe_82_Si_3.6_B_12.4−x_C_x_Cu_1_Ni_1_(x = 3, 4 and 5) amorphous ribbons: (**a**) XRD patterns; (**b**) DSC heat-flow curves at 20 K/min (exotherm downward); traces are vertically offset for clarity (scale bar: 1 W/g); arrows denote onset crystallization temperatures (*T*_x_); (**c**) Evolution of normalized loss modulus as a function of temperature, measured at 1 Hz with a heating rate of 5 K/min. The data are normalized by the maximum loss modulus ($E_{max}^{\prime \prime }$) to facilitate direct comparison of relaxation peak intensities among different samples; (**d**) variation in microhardness with C content at room temperature.

Figure 1a presents the XRD patterns of Fe_82_Si_3.6_B_12.4−x_C_x_Cu_1_Ni_1_ (x = 3, 4 and 5) amorphous ribbons. The absence of sharp Bragg peaks across all compositions confirms a fully amorphous structure, with no crystalline carbon-containing phases detected within the XRD detection limit. The DSC curves presented in Figure 1b illustrate the method of determining onset crystallization temperatures (*T*_x_) via extrapolation. It is evident that no clear glass transition peaks appear, which is characteristic of iron-based amorphous alloys. With increasing carbon content from C3 to C5, *T*_x_ decreases from approximately 663 K to 650 K. The ~800 K exotherm in Figure 1b is attributed to secondary crystallization—that is, the precipitation and growth of Fe–B–rich intermetallics (e.g., Fe_3_B/Fe_23_B_6_ or Fe(B, P), depending on composition)—occurring after the lower-temperature primary nanocrystallization of *α*-Fe(Si)/Fe_3_Si. This two-step pathway is well documented for Fe–Si–B–based (including FINEMET-type) glasses, with a first DSC exotherm associated with *α*-(Fe,Si)/Fe_3_Si formation and a second exotherm associated with Fe–B borides [33,34,35].

Figure 1c shows the variation in the normalized loss modulus $E^{\prime \prime }/E_{max}^{\prime \prime }$ as a function of temperature, where $E_{max}^{\prime \prime }\;$ represents the loss modulus at the α relaxation peak. When examining the relaxation behavior from low to high temperatures, two distinct relaxation peaks become evident. At lower temperatures (~490–500 K), the *β*-relaxation peak emerges, which corresponds to atomic activation processes within localized liquid-like regions [25,26]. The intensity of *β*-relaxation is directly linked to the plastic deformation capability of amorphous materials at room temperature, with a stronger *β*-relaxation peak typically indicating enhanced plasticity [1,4,26,36]. Additionally, *β*-relaxation is closely associated with structural heterogeneity [32,37], crystallization behavior, and soft magnetic properties [38]. At higher temperatures (~660 K), the *α*-relaxation peak appears, reflecting irreversible structural rearrangements due to large-scale atomic mobility [1,2]. This relaxation is intimately related to the glass transition, crystallization kinetics, and high-temperature plastic yielding behavior of amorphous alloys [2,28]. With increasing carbon from 3 to 5 at. %, the *β*-relaxation peak intensifies and shifts to lower temperatures, consistent with a higher fraction of liquid-like domains; prior studies directly link *β*-relaxation to nanoscale, loosely packed, low-modulus regions in metallic glasses [12,20,25,26,27,31,36,37,39,40]. This trend aligns with hardness measurements, where the C5 sample exhibits the lowest hardness among the studied compositions (Figure 1c). The peak temperature of *α*-relaxation decreases as the carbon content increases, suggesting that carbon addition enhances crystallization. This trend is consistent with the reduction in the onset crystallization temperature (*T*_x_) observed in the DSC curves (Figure 1b), further confirming that micro-alloying with carbon increases the activity of the glassy state and promotes crystallization.

### 3.2. Nanoindentation Creep

**Figure 2:**
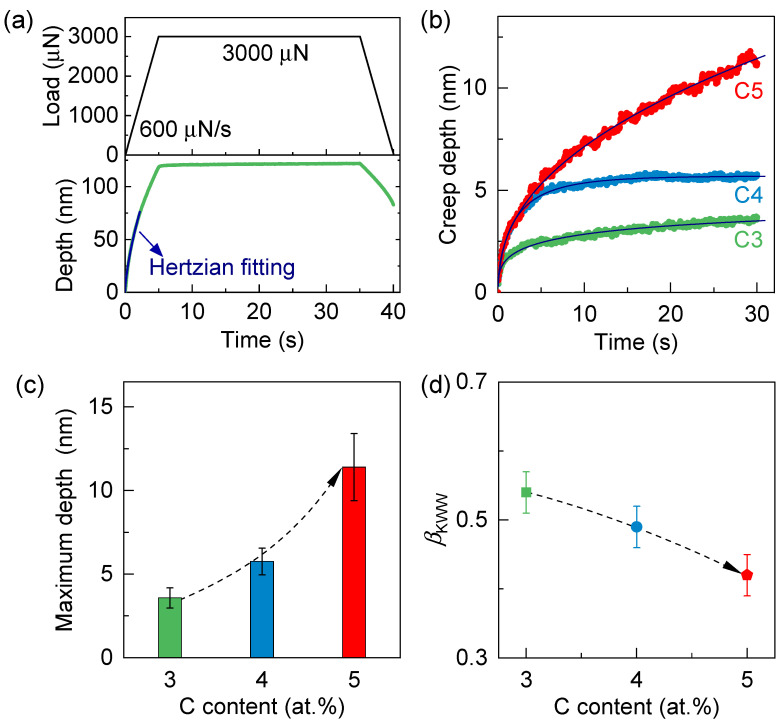
(**a**) Top: Schematic of the nanoindentation protocol, including loading ($\dot{P}=600\;\mu N/s$), holding at a constant peak load (*P* = 3000 μN) for 30 s, and unloading ($\dot{P}=-600\;\mu N/s$). Bottom: Corresponding indentation depth as a function of time. The blue curve represents the theoretical prediction based on Hertzian contact mechanics in the elastic regime. Deviations from this curve indicate the onset of plastic deformation. (**b**) Creep displacement profiles for Fe_82_Si_3.6_B_12.4−x_C_x_Cu_1_Ni_1_ amorphous ribbons with varying carbon content (x = 3, 4, 5). (**c**) Variation in maximum creep depth with increasing carbon content. (**d**) Stretching exponent $\beta_{KWW}$, indicative of dynamic heterogeneity, plotted as a function of carbon content.

To investigate the effect of micro-alloying on room-temperature plastic behavior, nanoindentation creep tests were performed under a constant peak load of 3000 μN held for 30 s, as shown in Figure 2a. The corresponding depth evolution with time is shown at the bottom of Figure 2a. In the initial elastic regime, the smooth depth-time response follows Hertzian contact theory:

(1) $$P\left(t\right)=\frac{4}{3}E_{r}\sqrt{R_{tip}}{h\left(t\right)}^{\frac{3}{2}}$$

where $R_{tip}$ represents the tip radius and $E_{r}$ represents the reduced elastic modulus [41]. Under the experimental loading rate of $\dot{P}=600\;\mu N/s$, the indentation depth as a function of time can be expressed as:

(2) $$h\left(t\right)={\left(\frac{3\dot{P}t}{4E_{r}\sqrt{R_{tip}}}\right)}^{\frac{2}{3}}$$

as indicated by the Hertzian fitting (blue curve) [41,42,43]. Figure 2b shows the creep displacement profiles for Fe_82_Si_3.6_B_12.4−x_C_x_Cu_1_Ni_1_ (x = 3, 4, and 5 at. %) amorphous ribbons. Because the specimens are free-standing, melt-spun ribbons rather than ribbons on a rigid substrate, any substrate effect can only originate from the finite ribbon volume beneath the indentation plastic zone [44,45]. With a very small depth-to-thickness ratio (≈120 nm/30 μm=1/250 = 0.4%), well below the 10% guideline, this influence is suppressed; accordingly, the extracted hardness and creep metrics are effectively insensitive to substrate bias under our testing conditions [46]. As the carbon content increases from 3% to 5%, the time-dependent maximum creep depth also increases, as summarized in Figure 2c. This trend suggests that carbon micro-alloying enhances plastic flow at room temperature, consistent with previous reports on ternary Fe-P-C bulk metallic glasses [9].

The enhanced creep deformation can be attributed to the intrinsic structural heterogeneity of metallic glasses. Due to the absence of long-range order, amorphous alloys exhibit a heterogeneous structure composed of densely packed solid-like regions and loosely packed, liquid-like regions [1,12,20,36]. The latter are characterized by higher atomic mobility and serve as the origin of shear transformation zones (STZs), which mediate plastic flow [2,12,31,32]. From a dynamic perspective, this heterogeneity can be quantified by the stretching exponent $\beta_{KWW}$ in the Kohlrausch–Williams–Watts (KWW) model, expressed as [12,32]:

(3) $$h=h_{0}\left[1-exp\left(-{\left(\frac{t}{\tau }\right)}^{\beta_{KWW}}\right)\right]$$

where $h_{0}$ is the creep amplitude, τ is the characteristic relaxation time, and $\beta_{KWW}$ describes the breadth of the relaxation time distribution [12,32]. A larger $\beta_{KWW}$ reflects a narrower distribution of relaxation times, indicative of a more homogeneous glassy structure [1,2]. As shown in Figure 2a, the creep curves for all samples were well fitted using the KWW model, with the extracted $\beta_{KWW}$ values summarized in Figure 2d. Notably, $\beta_{KWW}$ systematically decreases with increasing carbon content (from 3% to 5%), suggesting that higher carbon levels lead to enhanced dynamic heterogeneity in the amorphous structure. To quantify the rate, we evaluated a quasi-steady indentation creep rate from the existing constant-load hold, where the instantaneous rate is [47]:

(4) $$\dot{\varepsilon_{i}}\left(t\right)=\frac{1}{h\left(t\right)}\frac{dh}{dt}$$

and the quasi-steady value over 20–30 s is approximated by $\dot{\varepsilon_{i}^{\mathrm{ss}}}\approx \frac{1}{h\left(25\;s\right)}\;\frac{h\left(30\;s\right)-h\left(20\;s\right)}{10\;s}$. From the creep curves, we obtain $\dot{\varepsilon_{i}^{\mathrm{ss}}}\left(C3\right)\approx 1.4\times 10^{-3}\;s^{-1}$, $\dot{\varepsilon_{i}^{\mathrm{ss}}}\left(C4\right)\approx 1.8\times 10^{-3}\;s^{-1}$, and $\dot{\varepsilon_{i}^{\mathrm{ss}}}\left(C5\right)\approx 1.9\times 10^{-2}\;s^{-1}$. These values indicate an increase of roughly one order of magnitude from C3, C4 to C5, consistent with the carbon-induced strengthening of *β*-relaxation, a lower $\beta_{KWW}$, and a larger liquid-like fraction.

### 3.3. Mapping the Viscoelastic Heterogeneity

**Figure 3:**
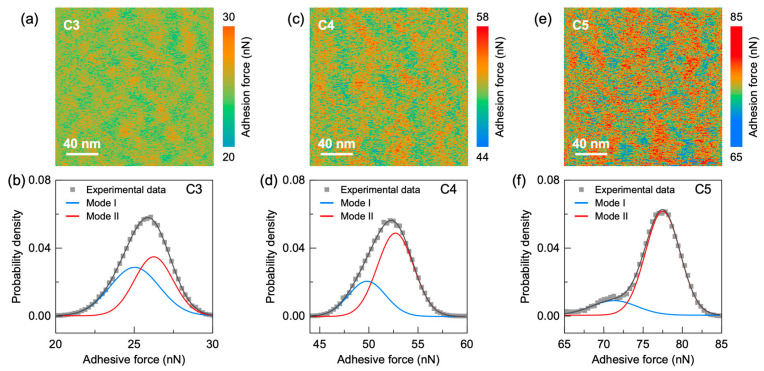
Local adhesion maps ((**a**,**c**,**e**); top) and corresponding probability-density functions ((**b**,**d**,**f**); bottom) for C3–C5. Bimodal Gaussian fits separate Mode I (solid-like) and Mode II (liquid-like) populations; contours highlight representative liquid-like clusters (high adhesion). Color bars (nN) match the right-hand legends and reflect each sample’s dynamic range (min–max indicated), while spatial scale bars (nm) are identical across panels.

To evaluate the evolution of nanoscale structural heterogeneity with carbon content, we mapped local adhesion by AFM (Peak Force-QNM) across Fe_82_Si_3.6_B_12.4−x_C_x_Cu_1_Ni_1_ (x = 3, 4 and 5) amorphous ribbons (Figure 3). The 40 nm-scale adhesion maps show pronounced spatial fluctuations in all samples, evidencing intrinsic nanoscale heterogeneity. In this framework, high-adhesion pixels correspond to more compliant, loosely packed, liquid-like regions with greater atomic mobility, whereas low-adhesion pixels mark denser, solid-like domains [27,30,31,32,37]. Quantitative comparison shows that the C5 sample exhibits the broadest adhesion force range (~20 nN), followed by C4 (~14 nN) and C3 (~10 nN), indicating that higher carbon content amplifies viscoelastic heterogeneity. To further analyze this trend, the corresponding probability density functions of adhesion force are shown in the lower panel of Figure 3. The distributions exhibit notable asymmetry and are well fitted by a bimodal Gaussian function. According to the flow unit model [12,30]. Mode I (blue) corresponds to solid-like regions, while Mode II (red) reflects liquid-like regions. With increasing carbon content, the distribution shifts toward higher adhesion values and the relative fraction of Mode II increases, suggesting a growing population of liquid-like regions. This microstructural change aligns with the observed reduction in hardness (Figure 1d), confirming that carbon micro-alloying induces softening by enhancing the population and distribution of liquid-like zones.

### 3.4. Physical Origins of Micro-Alloying in Promoting Plastic Flow

**Figure 4:**
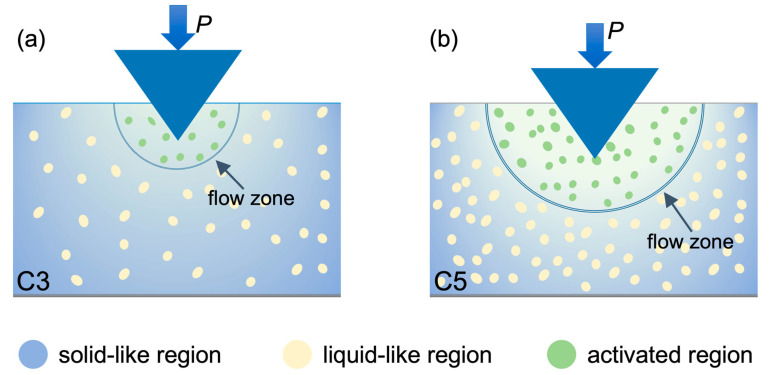
Schematic (not to scale) of local material response during nanoindentation creep for (**a**) C3 and (**b**) C5. *P* is the constant applied load. The blue background denotes the solid-like matrix; yellow dots are liquid-like domains; green dots mark mechanically activated regions within the indicated flow zone that participate in cooperative STZ-mediated plastic flow during the 30 s hold at P=3000 μN. The schematic qualitatively illustrates the larger activated fraction/flow-zone extent in C5 inferred from the stronger *β*-relaxation, broader AFM adhesion distributions, and greater creep depth.

Figure 4 presents a qualitative, not-to-scale schematic of the indentation geometry and the relatively larger population of mechanically activated domains in C5, which explains the observed plastic deformation during nanoindentation of Fe_82_Si_3.6_B_12.4−x_C_x_Cu_1_Ni_1_(x = 3, 4 and 5) amorphous ribbons. With increasing carbon content, the spatial distribution of liquid-like regions embedded within the solid-like matrix evolves toward greater complexity and heterogeneity. These loosely packed liquid-like domains, characterized by enhanced atomic mobility, serve as preferred sites for shear transformation zones (STZs), the fundamental carriers of plastic deformation in metallic glasses [1,20,48,49]. The carbon-rich alloy (C5) exhibits a more pronounced *β*-relaxation peak and reduced hardness (Figure 1), indicating a higher fraction and enhanced activity of liquid-like regions within the amorphous matrix. Residual stresses can alter structural heterogeneity and the strength/position of the *β*-relaxation (e.g., pressure shifts the *β* spectrum [50,51]). However, because our three compositions were processed identically with comparable wheel speeds and cooling histories, any process-induced residual-stress differences should be minimal and unlikely to account for the observed systematic trends [52,53].

The composition dependence of creep is quantitatively consistent with increased dynamic heterogeneity. From C3 to C5, constant-load creep depth rises while the KWW stretching exponent (*β*_KWW_) falls, indicating broader relaxation–time distributions; in parallel, the *β*-relaxation intensifies and shifts to lower temperature, and adhesion-force maps broaden with a larger liquid-like fraction. These trends yield consistent correlations between creep depth and (i) *β*_KWW_, (ii) *β*-relaxation metrics, and (iii) the liquid-like fraction. Within an STZ framework—where *β*-relaxation stems from localized rearrangements sharing activation barriers with STZ initiation—a higher density of loosely packed, low-modulus regions shortens local relaxation times and lowers activation barriers, facilitating STZ nucleation and cooperative shear, thereby enhancing room-temperature creep [12,20,25,26,27,31,36,37,39,40]. We therefore attribute the composition-induced creep increase to carbon-tuned structural/dynamic heterogeneity in Fe-based amorphous alloys.

## 4. Conclusions

Compositionally tuning Fe_82_Si_3.6_B_12.4−x_C_x_Cu_1_Ni_1_ (x = 3, 4, 5 at. %) clarifies the linkage from composition to dynamic *β*-relaxation and microstructural heterogeneity, and ultimately to mechanical response. Increasing carbon strengthens the β-relaxation, shifts its peak to lower temperature, and enlarges the fraction of liquid-like regions in nanoscale mapping which is accompanied by lower hardness, indicating enhanced local mobility and broader viscoelastic heterogeneity. Dynamic heterogeneity scales with creep: stronger *β*-relaxation, a larger liquid-like fraction, and a broader relaxation–time spectrum (smaller *β*_KWW_) each correlate with greater nanoindentation creep depth and rate under identical loading. These composition–response relationships provide a practical lever to tune time-dependent deformation and improve ductility in Fe-based amorphous alloys.

## Data Availability

The original contributions presented in this study are included in the article/Supplementary Materials. Further inquiries can be directed to the corresponding authors.

## Supplementary Materials

The following supporting information can be downloaded at: https://www.mdpi.com/article/10.3390/ma18194637/s1, Figure S1: Workflow of the experimental study: (1) alloy weighing and melting before casting (re-melt ×5 to ensure homogeneity) at *T*_melt_ ≈ 1200 °C (2) single-roller melt spinning (Cu wheel, 55 m/s; ribbons/films ≈ 30 μm thick) → (3) surface preparation and calibration (polish/clean; indenter and AFM calibration) → (4) structural/thermal characterization (XRD, DSC, DMA) → (5) nanoindentation creep (protocol as in Section 2.2) and AFM PeakForce-QNM mapping (adhesion/modulus) → (6) data reduction and correlation (linking relaxation, heterogeneity, and creep).
