# Supplementary material for: Carbon Micro-Alloying Promotes Creep Flow via Enhanced Structural Heterogeneity in Fe-Based Amorphous Alloys
Source: Materials (Basel). 2025 Oct 9;18(19):4637. doi: 10.3390/ma18194637 (PMC12526352; doi:10.3390/ma18194637)
Supplement: Supplementary file 1 [file materials-18-04637-s001.zip › materials-3872708-supplementary.pdf]

Article

Carbon Micro-Alloying Promotes Creep Flow via Enhanced Structural Heterogeneity in Fe-Based Amorphous Alloys

Deyu Cao [1,2], Sishi Teng [2], Jiajie Lv [2], Xin Su [2], Yu Tong [2,]*, Mingliang Xiang [2,]*, Lijian Song [2], Meng Gao [2], Yan Zhang [2], Juntao Huo [2] and Junqiang Wang [2,]*

[1] School of Materials Science and Engineering, Zhejiang University of Technology, Hangzhou 310014, China; caodeyu@nimte.ac.cn

[2] Zhejiang Key Laboratory of Magnetic Materials and Applications, Ningbo Institute of Materials Technology and Engineering, Chinese Academy of Sciences, Ningbo 315201, China; tengsishi@nimte.ac.cn (S.T.); lvjiajie@nimte.ac.cn (J.L.); suxin@nimte.ac.cn (X.S.); songlj@nimte.ac.cn (L.S.); gaomeng@nimte.ac.cn (M.G.); yzhang@nimte.ac.cn (Y.Z.); huojuntao@nimte.ac.cn (J.H.)

* Correspondence: tongyu@nimte.ac.cn (Y.T.); xiangmingliang@nimte.ac.cn (M.X.); jqwang@nimte.ac.cn (J.W.)

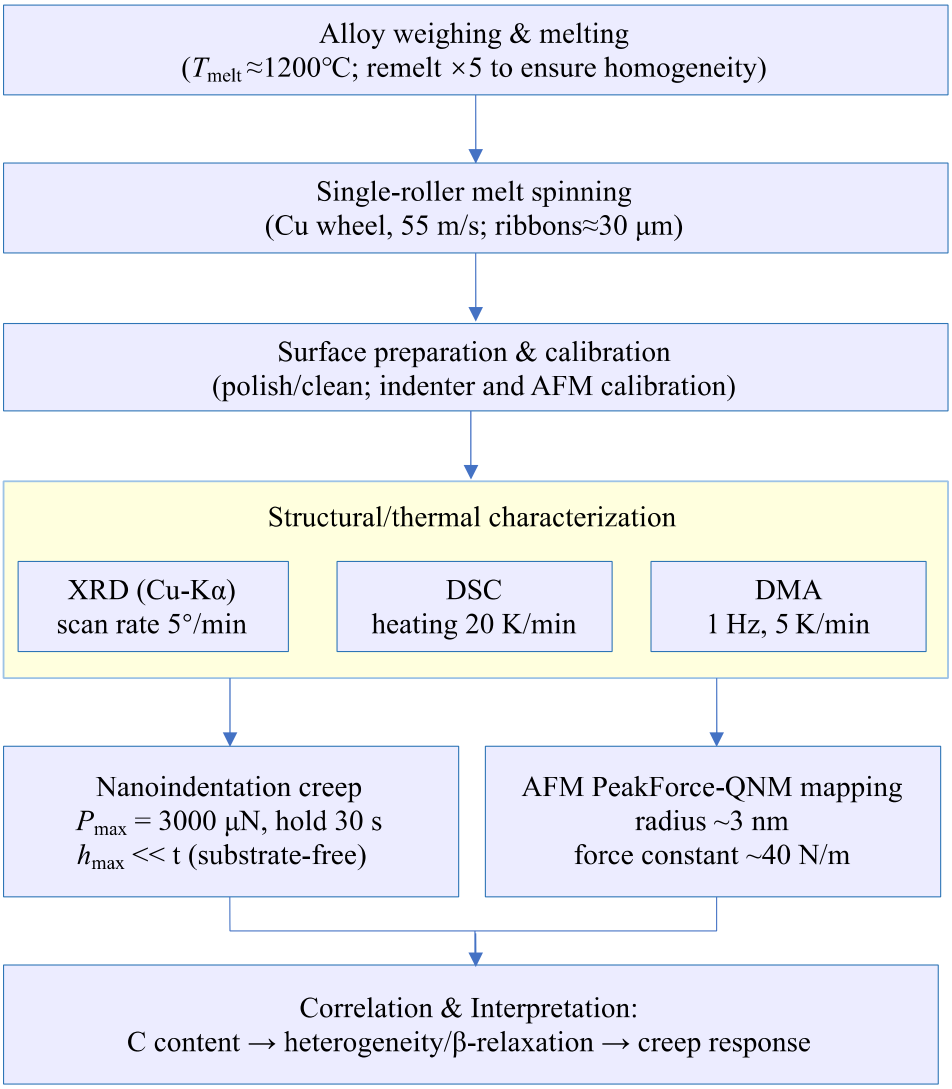

Figure S1. Workflow of the experimental study: (1) alloy weighing and melting before casting (re-melt ×5 to ensure homogeneity) at T_{melt}≈1200°C → (2) single-roller melt spinning (Cu wheel, 55 m/s; ribbons/films ≈ 30 μm thick) → (3) surface preparation and calibration (polish/clean; indenter and AFM calibration) → (4) structural/thermal characterization (XRD, DSC, DMA) → (5) nanoindentation creep (protocol as in Section 2.2) and AFM PeakForce-QNM mapping (adhesion/modulus) → (6) data reduction and correlation (linking relaxation, heterogeneity, and creep).
